# A Constitutively Active Gαi3 Protein Corrects the Abnormal Retinal Pigment Epithelium Phenotype of *Oa1−/−* mice

**DOI:** 10.1371/journal.pone.0076240

**Published:** 2013-09-30

**Authors:** Alejandra Young, Ying Wang, Novruz B. Ahmedli, Meisheng Jiang, Debora B. Farber

**Affiliations:** 1 Jules Stein Eye Institute, University of California Los Angeles, Los Angeles, California, United States of America; 2 Molecular Biology Institute, University of California Los Angeles, Los Angeles, California, United States of America; 3 Brain Research Institute, University of California Los Angeles, Los Angeles, California, United States of America; 4 Department of Molecular and Medical Pharmacology, University of California Los Angeles, Los Angeles, California, United States of America; National Eye Institute, United States of America

## Abstract

**Purpose:**

Ocular Albinism type 1 (*OA1*) is a disease caused by mutations in the *OA1* gene and characterized by the presence of macromelanosomes in the retinal pigment epithelium (RPE) as well as abnormal crossing of the optic axons at the optic chiasm. We showed in our previous studies in mice that Oa1 activates specifically Gαi3 in its signaling pathway and thus, hypothesized that a constitutively active Gαi3 in the RPE of *Oa1−/−* mice might keep on the Oa1 signaling cascade and prevent the formation of macromelanosomes. To test this hypothesis, we have generated transgenic mice that carry the constitutively active Gαi3 (Q204L) protein in the RPE of *Oa1−/−* mice and are now reporting the effects that the transgene produced on the *Oa1−/−* RPE phenotype.

**Methods:**

Transgenic mice carrying RPE-specific expression of the constitutively active Gαi3 (Q204L) were generated by injecting fertilized eggs of *Oa1−/−* females with a lentivirus containing the Gαi3 (Q204L) cDNA. PCR, Southern blots, Western blots and confocal microscopy were used to confirm the presence of the transgene in the RPE of positive transgenic mice. Morphometrical analyses were performed using electron microscopy to compare the size and number of melanosomes per RPE area in putative *Oa1−/−,* Gαi3 (Q204L) transgenic mice with those of wild-type NCrl and *Oa1−/−* mice.

**Results:**

We found a correlation between the presence of the constitutively active Gαi3 (Q204L) transgene and the rescue of the normal phenotype of RPE melanosomes in *Oa1−/−,* Gαi3 (Q204L) mice. These mice have higher density of melanosomes per RPE area and a larger number of small melanosomes than *Oa1−/−* mice, and their RPE phenotype is similar to that of wild-type mice.

**Conclusions:**

Our results show that a constitutively active Gαi3 protein can by-pass the lack of Oa1 protein in *Oa1−/−* mice and consequently rescue the RPE melanosomal phenotype.

## Introduction

X-linked ocular albinism is a disorder of melanosome biogenesis leading to congenital visual impairment in males [1]. Affected individuals exhibit nystagmus, reduced visual acuity, hypopigmentation of the iris and retinal pigment epithelium (RPE), foveal hypoplasia, ocular misrouting, reduced or absent binocular functions, photoaversion, strabismus and giant melanosomes in the RPE and skin melanocytes [2]. In addition to macromelanosomes, *OA1* patients (as well as *Oa1* knockout mice) have a reduction of ipsilateral retinal ganglion axons at the optic chiasm. These are the two main phenotypic characteristic of ocular albinism [3]. Mutations in the *OA1* gene [4], also known as the *GPR143* gene, are responsible for this disease. More than 60 mutations (missense, nonsense, frameshift or splice-site mutations) have been identified in affected individuals [4], [5]. These changes result in a nonfunctional OA1 protein and often prevent it from reaching its normal location at melanosomal membranes [6], [7], [8] or from interacting with other molecules of its signaling pathway [9]. Without a functional GPR143 protein, melanosomes in the RPE and melanocytes of the skin become abnormally large, but it is unclear how these macromelanosomes are related to vision abnormalities in patients with ocular albinism.

The OA1 protein is an intracellular G protein-coupled receptor localized in the RPE. We previously identified the inhibitory GTP-binding protein alpha subunit polypeptide 3 (Gαi3) as the specific downstream component of the Oa1 signaling cascade of mice. In addition, we showed that *Oa1* and *Gαi3* knockout mice present similar abnormal macromelanosomes in the RPE and misrouting of optic axons at the optic chiasm [10]. These findings strongly suggested a common Oa1-Gαi3 signaling pathway, supporting a previously unsuspected role for Gαi3 in the events directly or indirectly related with melanosomal biogenesis.

Gαi3 has been shown to regulate multiple pre- and post-Golgi trafficking steps, suggesting that it may function at variable sites across the Golgi stacks of different cells. In renal cells, Gαi3 is exclusively located on Golgi membranes [11], [12]. In exocrine pancreatic cells Gαi3 is found not only in *cis*- and *trans*-Golgi membranes, but also on vesicles at both sides of the Golgi stack [13]. In addition, Gαi3 has been demonstrated to regulate protein trafficking in a variety of pathways, i.e., in human intestinal cells, human colon cancer cell line HT-29, human epithelial cells LLC-PK1/NRK and murine erythroleukemia (MEL) cells [12], [14], [15], [16], [17], [18]. Moreover, Gαi3 has been suggested to have a role in macroautophagy in the colon carcinoma cell line HT-29 [17] and a role in the insulin-mediated regulation of autophagy [19].

While the precise function of Gαi3 in RPE melanosome biogenesis remains to be delineated, our studies suggest that this protein plays an important role in the control of the size and density of RPE melanosomes and therefore in the regulation of RPE pigmentation [10], [20]. We hypothesize that a constitutively active Gαi3 protein could by-pass the lack of Oa1 in *Oa1−/−* mice and keep the Oa1 signaling cascade going leading to the normalization of their RPE pigmentation. In this study we show that, indeed, introducing the constitutively active Gαi3 (Q204L) protein as a transgene in *Oa1−/−* mice rescues in them the RPE melanosomal phenotype characteristic of wild-type mice.

## Materials and Methods

### Transgenic Construct

**Figure 1:**
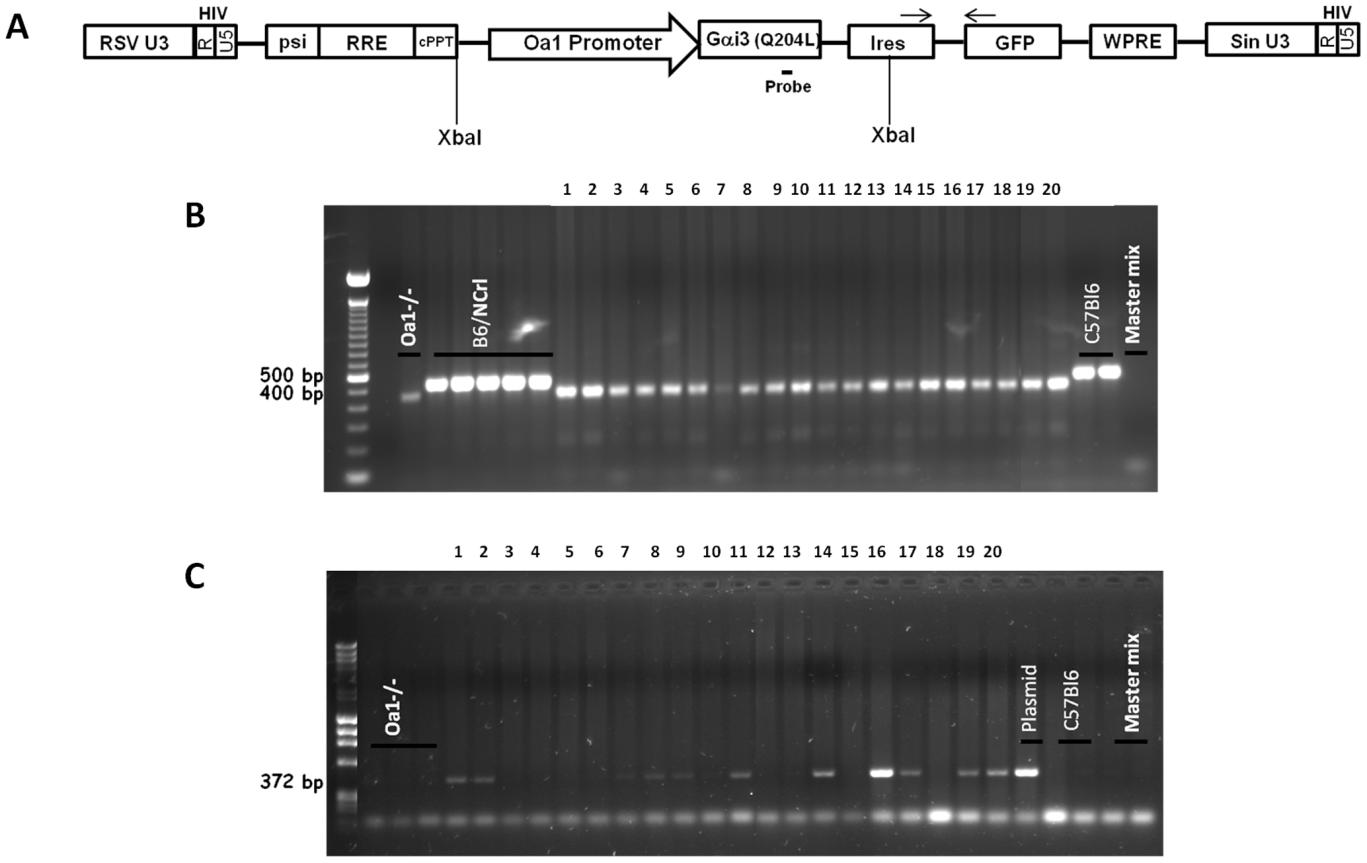
A: Identification of *Oa1−/−,* Gαi3 (Q204L) transgenic mice. The lentivirus construct used to generate *Oa1−/−,* Gαi3 (Q204L) transgenic mice contains the constitutively active Gαi3 (Q204L) cDNA driven by the *Oa1* promoter and ires-GFP. Abbreviations: RSV, Rous sarcoma virus promoter; U3-HIV-R-U5, 5′ and 3′ detailed long terminal repeats (LTRs); psi, packaging signal for viral RNA into virus capsids to continue the infection of HIV in its host; RRE, Rev-responsive element; cPPT, central polypurine tract; *Oa1* (ocular albinism type 1) promoter; Gαi3 (Q204L), constitutively active Gαi3 cDNA; ires, internal ribosome entry site; GFP, green fluorescence protein; WPRE, Woodchuck hepatitis virus post-transcriptional regulatory element; sin U3, self-inactivating element that relies on the introduction of a deletion in the U3 region of the 3′ (LTR). *XbaI,* restriction enzyme site. Two short thin black arrows indicate the forward and reverse ires-GFP primers used for PCR amplification. The probe used for Southern blots binds to Gαi3 (Q204L) cDNA at the position indicated in the figure. **B:** 1.8% agarose gel showing PCR analysis of the *Oa1* gene in putative founder transgenic mice. Specific primer sets (HPRT and *Oa1*) were used to amplify a 400 bp band of the HPRT cassette and a 500 bp of the endogenous *Oa1* gene, respectively. The 20 putative transgenic founders analyzed on the gel had the 400 bp band, indicating that all animals were generated in the *Oa1−/−* background. **C:** Identification of positive transgenic founders by PCR. 1.8% agarose gel identifying positive Gαi3 (Q204L) transgenic mice. Genotyping was done using specific primers that amplify a 372 bp fragment between the ires and GFP regions of the transgenic construct. Controls used: *Oa1−/−*, NCrl, C57Bl/6 genomic DNA samples and transgenic plasmid. The Master mix was loaded as a control for contamination.

The expression vector containing the active mutant Gαi3 (Q204L) under the control of the *Oa1* promoter was constructed by subcloning a 3.5 kb genomic DNA fragment of the mouse *Oa1* promoter region, a 1.1 kb human Gαi3 cDNA fragment encoding the Q204L mutation, and a 0.5 kb polyadenylation signal sequence into the pKS pBluescript II KS+ plasmid (Clontech, Palo Alto, CA). The ∼5.1 kb *NotI-XbaI Oa1*-Gαi3 (Q204L) fragment was cloned into the *BamHI* and *PstI* sites of a VSVG pseudotyped lentivirus vector. The vector also contained an ires-GFP sequence downstream of the *Oa1*-Gαi3 (Q204L) cassette. Both ends of the insert were sequenced to ensure that the joining was correct. The final lentivirus expression vector (Figure 1A) was packed in 293T cells. The titer of the virus was determined by measuring the viral capsid p24 protein. There are approximately 1×10^4^ particles of lentivirus for every pg of p24 antigen, which works out to be 100 transducing units of virus for every pg of p24 antigen present (Sigma Aldridge protocol). The virus packing and production were done at the UCLA Vector Core Facility.

### Ethics Statement

All experiments involving mice were carried out using protocols approved by the UCLA Animal Research Committee, and in accordance with the ARVO Statement for Use of Animals in Ophthalmic and Vision Research.

### Generation of transgenic mice carrying the constitutively active Gαi3

Wild-type C57Bl/6NCrl (hereafter NCrl) mice and congenic *Oa1* knock-out mice (*Oa1−/−*) were obtained from The Charles River Labs, USA and Italy, respectively. Mice were housed and bred in conventional cages and environmental conditions at the UCLA animal facility. Transgenic animals were generated by microinjection of lentivirus to embryos at the UCLA Transgenic Core Facility. Briefly, fertilized eggs at the pronuclear stage from *Oa1−/−* mice were microinjected with the lentivirus (∼1 picoliter, equivalent to the size of nuclei) containing the constitutively active human Gαi3 (Q204L) cDNA. The injected eggs were then implanted into surrogate mothers to complete the gestation. These putative *Oa1−/−*, Gαi3 (Q204L)-ires-GFP transgenic mice were tagged, genotyped by PCR and used to expand the colony.

### Genotyping of transgenic mice

Genomic DNAs were extracted from tail biopsies with Protease K digestion and phenol/chloroform extraction. Mouse genotyping was carried out by PCR and used to confirm the *Oa1−/−* background and to determine the presence of the Gαi3 (Q204L) transgene in their genome. To corroborate the absence of the *Oa1* gene, the *Oa1* primer set (Table 1) was used to produce a 500 bp fragment representative of the wild-type *Oa1*+/+ allele. In addition, the HPRT primer set was used to produce a 400 bp product (Table 1). The HPRT band is a distinct feature of the knockout *Oa1 −/−* mice. PCR reactions were performed in a total volume of 25 µl [2.5 µl of 10X PCR buffer, 2 µl of dNTPs (each 2.5 mM), 1 µl of each primer (10 pmol/µl each), 0.2 µl of Taq polymerase (1U), and ddH_2_O to 25 µl], for a total of 30 cycles using the following conditions: denaturing at 94°C for 30 sec., annealing at 55°C for 45 sec. and extension at 72°C for 45 sec. To determine the presence of the Gαi3 (Q204L) transgene in the mice genome, the ires-GFP region of the lentivirus construct was amplified. The ires-GFP primer set was used to produce a 372 bp DNA fragment. The PCR reaction was set up as above and run using a total of 33 cycles of denaturing at 94°C for 30 sec., annealing at 62°C for 1 min. and extension at 72°C for 2 min. All samples were run on 1.8% agarose gels.

**Table:** Table1. Primer sequences (5′ to 3′) used for PCR and Southern blot genotyping.

Primer set	Forward	Reverse	Product Size (bp)
HPRT	taagttctttgctgacctgct	ggctttgtatttgccttttcc	400
*Oa1*	acatgacgcccaatctccctc	tagactaccctctgagtccag	500
ires-GFP	tgaccctgaagttcatctgca	ttcttctgcttgtcggcggtg	372
Gαi3 (Q204L)Exon 6-7	gtttgatgtaggtggcctaag	ggataacagatagttaacgga	272

### Southern blot

Genomic DNA from each mouse (10 µg) was digested with *XbaI* separated by electrophoresis on a 1% agarose gel and transferred onto Hybond-N+ membrane (Amersham Biosciences, NJ). The hybridization probe was generated by PCR using the Gαi3 (Q204L) primer set designed to amplify a sequence of the Gαi3 (Q204L) cDNA that corresponds to the end of exon 6 and beginning of exon 7 of the *Gαi3* gene. The gel purified-PCR product was then labeled with [α-^32^P] dCTP using Ready-To-Go DNA labeling beads (Amersham Biosciences). Presence of the transgene was confirmed by an expected 4.7 kb radioactive band.

### Morphometrical Analysis

3-month-old *Oa1−/−*, Gαi3 (Q204L)-ires-GFP positive and negative transgenic mice, NCrl and *Oa1−/−* mice were deeply anesthetized by Isoflurane (30% concentration) inhalation. The left eyes were enucleated for protein extraction before intravenous perfusion with 2% formaldehyde and 2.5% glutaraldehyde in 0.1 M sodium phosphate buffer, pH 7.4. After perfusion, the right eyes were enucleated, washed in 0.1 M sodium phosphate buffer, fixed with 1% osmium tetroxide, washed with double distilled cold water, dehydrated in ethanol solutions of increasing concentrations, and embedded in Epon/Araldite mixed resin.

#### Electron microscopy

The nasal regions of all eyes were sectioned into superior and inferior quadrants. Eye quadrants were rinsed in 0.1 M sodium phosphate buffer, post-fixed with 1% buffered osmium tetroxide, dehydrated in ethanol solutions of increasing concentrations, and embedded in araldite 502 (Electron Microscopy Sciences, PA). Sections (60–70 nm) were cut on a Leica Ultracut UCT and collected on 200 mesh uncoated copper grids. For the ultrastructural analysis, stained sections (5% uranyl acetate and 0.4% lead citrate) were analyzed with a 910 Zeiss electron microscope and the RPE fields were photographed using a Keenview™digital camera. For quantification of melanosomes and determination of their area in the RPE, we analyzed 12 micrographs corresponding to the nasal region of the eye at 16,000X magnification using the analysis™ software for LEO 900 TEM, version 3.2. (Soft Imaging System, Lakewood, CO). To determine the size and number of melanosomes, each micrograph was loaded into the Soft Imaging System analySIS software platform and the area of individual melanosomes was selected and measured with the magic wand tool. In addition, the total RPE area containing the melanosomes was selected and measured using the pencil tool. The program produced two spreadsheets per micrograph containing the melanosomal count, the individual areas, and their mean size. We analyzed the differences in the melanosomal size in the RPEs from the different eyes and the percent frequency of melanosomes in the 100 nm^2^ to more than 15,000 nm^2^ size range. We also calculated the number of melanosomes per RPE area in the transgenic mice and the controls NCrl and *Oa1−/−* mice. Comparisons among all groups were done using a one-way analysis of variance (ANOVA). Standard errors of the mean are shown in the statistical analysis.

### Western blot analysis

The GFP protein expression in RPE-Choroid protein extracts from one eye of each NCrl and *Oa1−/−* control mice and from five putative transgenic mice was determined using an anti-GFP antibody (Cell Signaling technology, Danvers, MA). Protein extracts (15 µg per lane) were separated by SDS-PAGE using a 4–12% gradient Tris-gel (Life Technologies, NY) and blotted onto nitrocellulose. The membranes were blocked in Odyssey's blocking buffer (Li-Cor Biotechnology, NE) and probed overnight at 4°C with primary polyclonal rabbit anti-GFP antibody diluted 1∶2,000 (Santa Cruz Biotechnology, Santa Cruz, CA), followed by incubation for 45 min at RT with secondary Alexa Fluor 680-conjugated goat anti-rabbit IgG diluted 1∶5,000. Antibody recognition was detected with an Odissey scanner according to the manufacturer’s instructions (Licor Biotech, NE).

### Confocal Microscopy

Eyes from 6 month-old mice positive for the transgene (lines 357 and 905) were enucleated, rinsed in 0.1 M sodium phosphate buffer, post-fixed with 4% paraformaldehyde, and embedded in OCT compound prior to cryostat sectioning. Cryostat sections were cut at 8 µm, mounted on histological slides, and air dried at room temperature for direct observation of GFP fluorescence and nuclei stained with DAPI. Images were taken using a 60X objective lens and a Zoom of 2.9 for a total magnification of 174X.

## Results

### Generation of *Oa1−/−*, Gαi3 (Q204L) transgenic mice

We generated transgenic mice using a VSVG pseudotyped lentivirus vector carrying the constitutively active Gαi3 (Q204L) protein expression cassette. Appropriate expression of this constitutively active Gαi3 in the RPE of *Oa1−/−* mice was obtained using the RPE-specific promoter of the *Oa1* gene (Figure 1A). It has been shown that transgenic mice can be generated efficiently by microinjection of lentivirus into the perivitelline space of single-cell mouse embryos, and that injecting a high dose of virus can produce near 100% transgenesis with variable copies of transgene in transgenic lines [21]. In this study, we intended to generate transgenic lines with one or few copies of the transgene by lowering the lentivirus input, aiming for a 40∼70% transgenesis rate. The titer of lentivirus used was 18.9 µg/ml of p24 protein, which was estimated to be 1.9×10^9^ transducing units/ml. ∼0.5–1pL of the stock virus, which is equivalent to ∼1–2 transducing units, were injected into the perivitelline space of single-cell mouse embryos. Embryos were implanted into pseudo-pregnant females and were carried to full term yielding 20 putative transgenic founder pups (tagged with IDs 1-20).

Initial genotyping verified that these pups were *Oa1* knockouts. The *Oa1−/−* mice used for these experiments had been generated by introducing an HPRT cassette to delete exon 1 of the *Oa1* gene [22]. PCR amplification of mouse genomic DNA using the HPRT cassette primer set (Table 1) produces a 400 bp band characteristic of the *Oa1−/−* allele. The *Oa1* wild-type primer set (Table 1), which amplifies a 500 bp fragment of the *Oa1* gene exon 1 was used to identify the *Oa1* wild-type allele. The DNA samples from all founder transgenic mice showed only the targeted allele 400 bp amplicon and lacked the wild-type 500 bp PCR band (Figure 1B), indicating that indeed, all 20 pups were derived from *Oa1−/−* mice.

Then, we genotyped the pups to identify transgenic founders by PCR analysis using the ires-GFP primer set (Table 1). Our results showed that 11 pups out of the 20 generated presented the expected 372 bp PCR band for the transgene while the other 9 pups were negative for the transgene (Figure 1C). Since we only injected few transducing units per each embryo, the transgenesis rate was expected to be 40∼70%, and positive transgenic founders were anticipated to contain one or two copies of the transgene. The PCR results showed the generation of 60% transgenic founders, which falls into the ballpark of our strategy. In addition, there was a variation in the intensity of the transgene PCR bands among the positive transgenic lines, which might reflect the different copy number. We grouped founder lines according to the intensity of the transgene PCR band normalized to the corresponding HPRT cassette amplicon intensity in the pups. The progeny group with lower intensity more likely represents that with a single copy of the transgene, while samples with strong intensity perhaps had two or more copies.

### Germline transmission of the Gαi3 (Q204L) transgene

**Figure 2:**
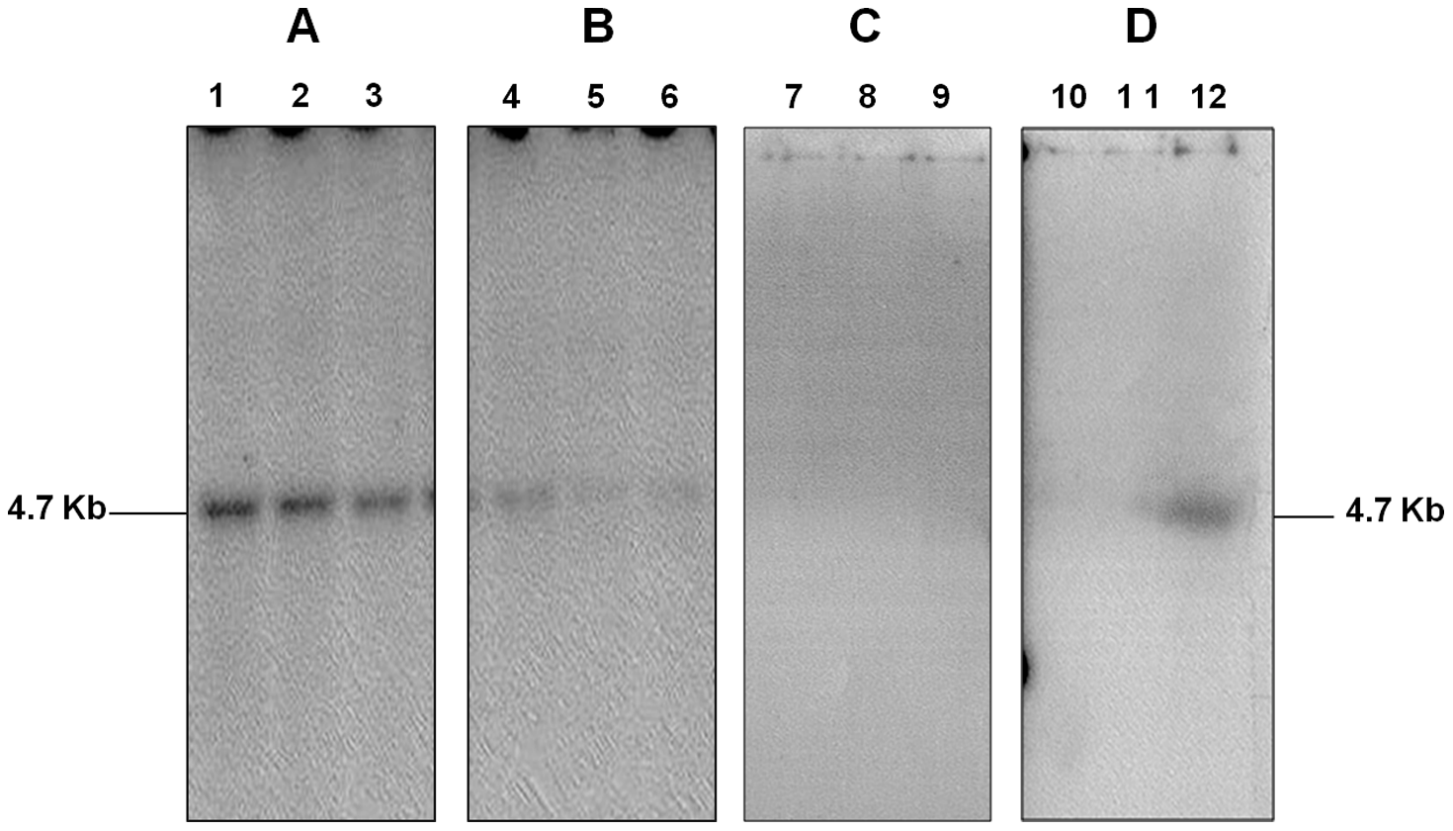
Southern blot analysis of transgenic progeny. Positive transgenic mice were identified by the presence of the expected 4.7 µg of mouse genomic DNA. **A.** Positive transgenic mice presenting a strong signal on the Southern blot: Lane 1: line 142. Lane 2: line 131. Lane 3: line 157. **B:** Positive transgenic mice with a weak signal: Lane 4: line 377. Lane 5: 374, Lane 6: line 396. **C:** Mice without integrated transgene show no radioactive signal: Lane 7: line 223. Lane 8: Line 275. Lane 9: line 276. **D**. Negative controls NCrl and line 13 (Lanes 10 and 11) and positive control transgenic mouse line 16 (Lane 12).

**Figure 3:**
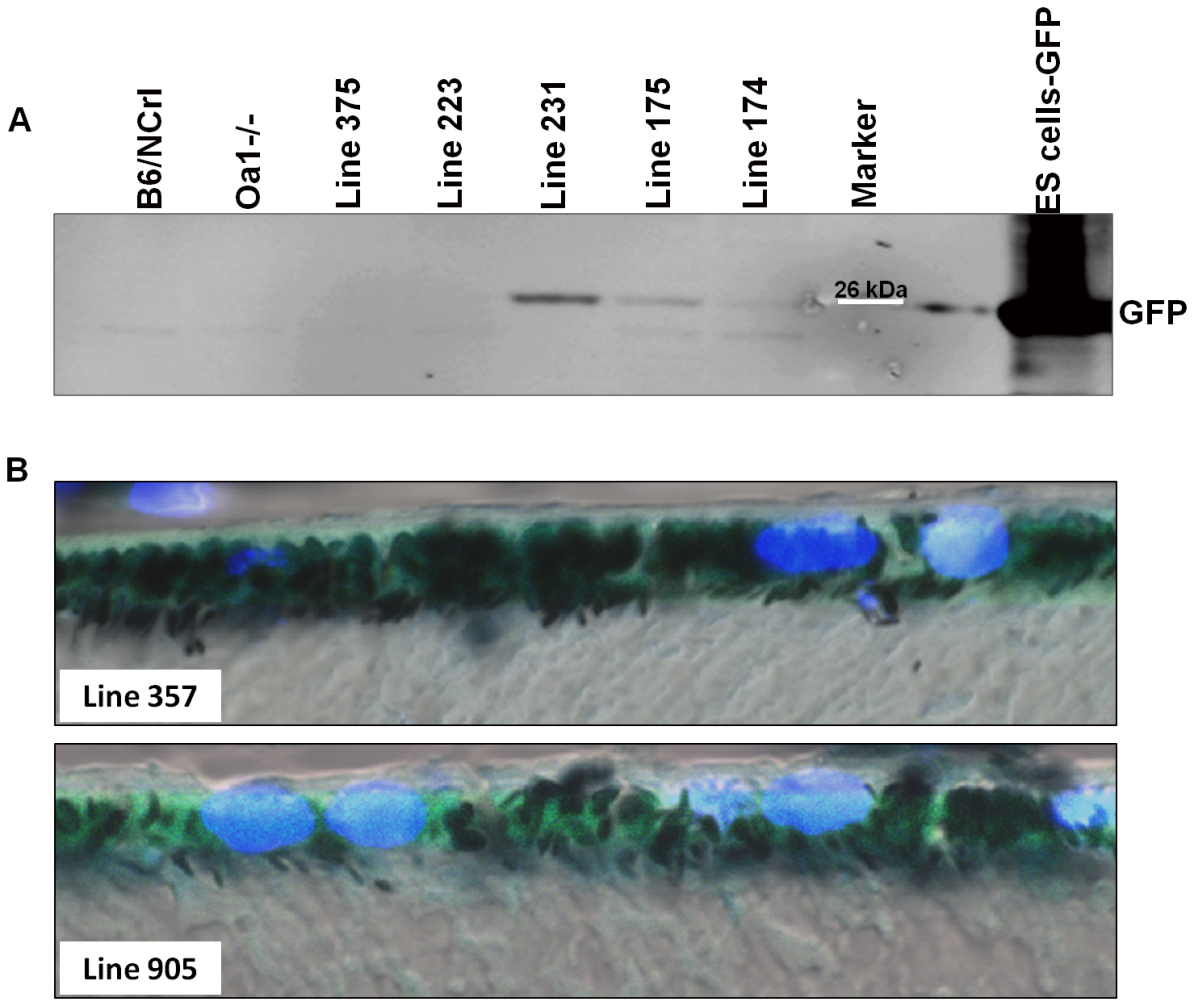
GFP expression in the RPE varies in different transgenic mice, suggesting a possible correlation between level of GFP expression and transgene copy number. A: Western blot using RPE/choroid protein extracts and GFP antibody. Positive transgenic mouse lines 231,175 and 174 expressed GFP in the RPE. Controls NCrl and *Oa1−/−*, as well as mice without integrated transgene (lines 375 and 223) did not express GFP. B: Confocal images (magnification: 174X) of the RPE from positive transgenic mice show expression of GFP.

The innate ability of lentiviral vectors to integrate into the host genome with high efficiency is limited by the fact that each lentiviral integration site contains a single copy of the transgene, rather than the head to tail arrays of multiple transgenes that occur when using DNA pronuclear injection. This means that multiple copies of the transgene will be segregated in the progeny. Therefore, we interbred the founders to generate a diverse group of offspring, having negative, single copy and multiple copies of the Gαi3 (Q204L) transgene. This breeding scheme could also help us to identify any false positive restoration phenotypes caused by the lentivirus random integration leading to the host genes inactivation. The resulting progeny and subsequent generations of mice were tagged with ID numbers 100–1000, without parental identification. Southern blots were used to determine the relative transgene expression level in fifty mice derived from the interbreeding of transgenic lines. Results of the Southern blots indicate that the transgenic mice progeny could be classified into three different groups (negative, low and high copy number) according to the relative hybridization intensity (Figure 2), similar to the different intensities of the PCR bands obtain from the founder transgenic mice, showed in Figure 1C. Among the progeny, the first group represents positive transgenic mice with a strong signal: lines 142, 131, 157, (Figure 2A, lanes 1–3, respectively). The second group corresponds to positive transgenic mice with weak radioactive band intensity: lines 377, 374, and 396 (Figure 2B, lanes 4–6, respectively). The last group shows negative transgenic mice with absence of radioactive band: lines 223, 275, and 276 (Figure 3C, lanes 7–9, respectively). We conclude that the transgene can effectively be transmitted to offspring and that we were able to obtain lines with different copy numbers.

### Specific RPE expression of GFP in transgenic lines

Since no highly specific anti-Gαi3 antibody is available, we designed the viral expression cassette with ires-GFP to monitor the expression of the transgene indirectly. We performed Western blot analysis to detect GFP expression in RPE cells from a single eye using anti-GFP antibodies. The 26-kDa GFP band was detected in the positive transgenic lines and absent in non-transgenic lines (Figure 3A). Variable intensity of the GFP signal was also observed among the positive lines. Line 231 showed strong staining intensity, while lines 174 and 175, had a weak signal. Lines 223 and 375 were negative for GFP. In addition, confocal microscopy of fixed, OCT embedded and mounted retina sections showed expression of GFP in the RPE of positive transgenic mice from lines 357 and 905 (Figure 3B). Thus, Western and confocal analyses of GFP expression in the RPEs of transgenic mice demonstrated that the transgene is stably integrated and transmitted through the germline and not silenced. Genotyping analyses (PCR and Southern) demonstrated a correlation of transgene copy number with the transgene protein expressed (Western).

### Rescue of the RPE phenotype in *Oa1−/−* mice by expression of the Gαi3 (Q204L) transgene

**Figure 4:**
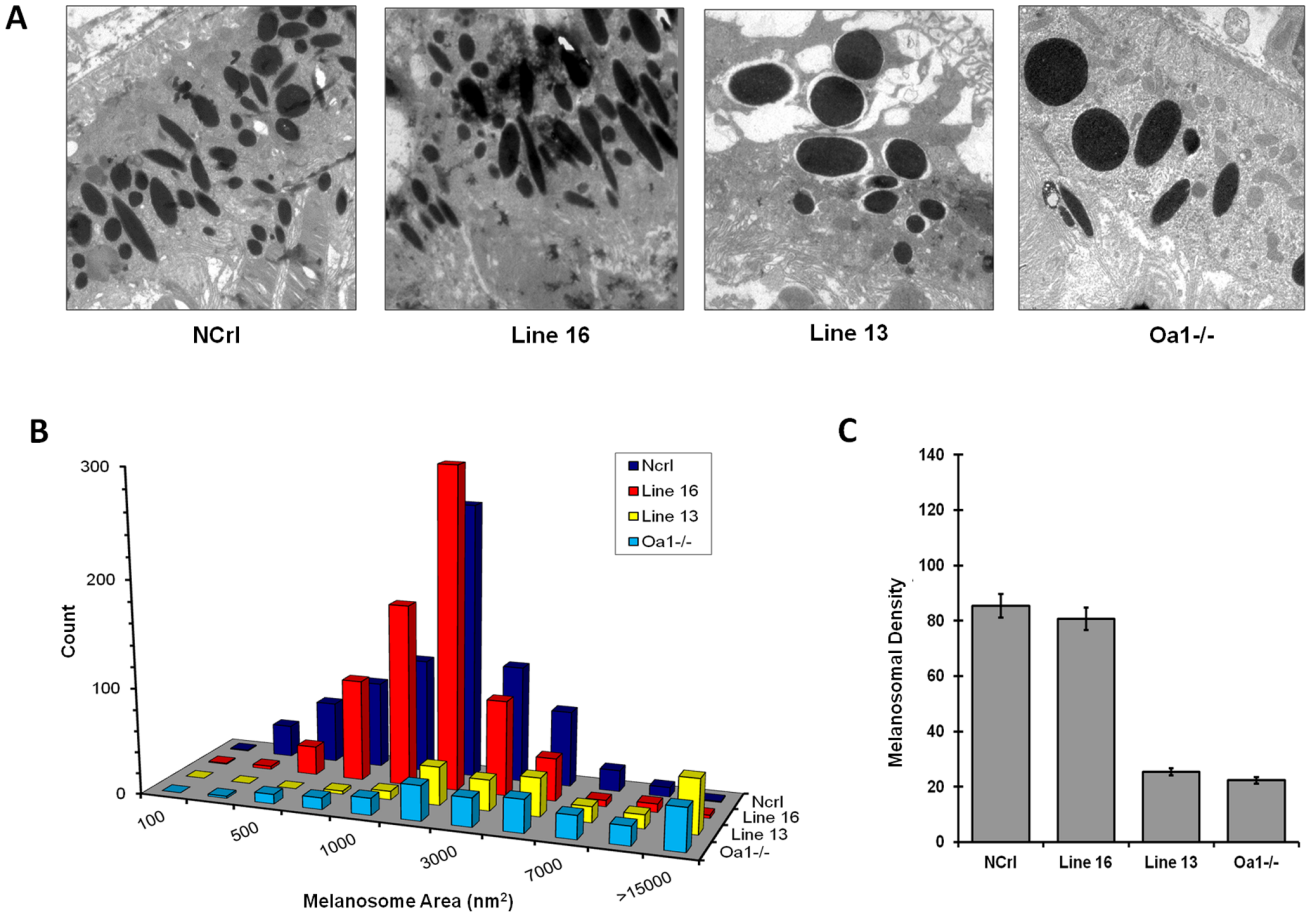
Electron microscopy and densitometry analysis of putative transgenic founder mice. A: Electron micrographs comparing RPEs from positive and negative transgenic founder mouse lines 16 and 13 to wild-type NCrl and *Oa1−/−* RPEs, respectively. Panel **A** shows electron micrographs of RPEs from: NCrl, transgenic line 16 and line 13 without integrated transgene and *Oa1−/−* mice. **B-C:** Morphometrical analysis of RPE melanosomes from lines 16 and 13. **B:** Histogram representing the percent distribution of melanosomes by size. RPEs from *Oa1−/−* mice and line 13 have highest percentage of melanosomes larger than 15,000 nm^2^ (19.1% and 26.7% respectively), while NCrl, and line 16 RPEs have higher frequency of smaller and medium size melanosomes (500–3000 nm^2^), 67.6% and 81.6%, respectively. **C.** *Oa1−/−* and line 13 RPEs have very low mean melanosomal densities, 22.3 and 25.3 melanosomes per RPE area, respectively. NCrl and line 16 RPEs have mean densities of 85.5 and 80.7 melanosomes per RPE area, respectively.

Lentiviral microinjection at high percentage of transgenesis rate allowed us to study directly the effect of RPE phenotype restoration by the constitutively active Gαi3 (Q204L) transgene in transgenic founders. To test whether expression of the active Gαi3 mutant in the RPE of *Oa1−/−* mice can bypass the requirement of a functional *Oa1* receptor, we performed morphometrical analysis of melanosomal size and density in the RPEs of transgenic mice using electron microscopy. We analyzed and compared the transgene line 16, which has several copies of the transgene, with the negative control line 13. The RPE of the positive transgenic founder line 16 did not present macromelanosomes, which are characteristic of the parental *Oa1−/−* mice; instead it had a homogeneous, smaller in size, melanosomal population similar to that observed in wild-type NCrl RPE (Figure 4, A and B). On the other hand, the RPE of negative transgenic mouse line 13 exhibited a similar *Oa1−/−,* abnormal, macromelanosomal phenotype (Figure 4, C and D). Accordingly, melanosomes 2,000 nm^2^ in size were present in high numbers in NCrl and line 16 RPEs (34.6% and 45.7% respectively) but were found at a lower frequency in *Oa1−/−* and negative transgenic line 13 RPEs (15.8% and 18.8%, respectively) (Figure 4B). In addition, our results indicate that the presence of the transgene in mice of the *Oa1−/−* background correlates with a high number of melanosomes per RPE area, similar to what is observed in wild-type NCrl RPE: i.e., positive transgenic mouse line 16 had a mean density of 80.7±12.7 melanosomes per RPE area, and wild-type NCrl of 85.5±6.5 (Figure 4C). Thus, these results suggest that the presence of the transgene could restore the normal melanosomal size and density in the *Oa1−/−* RPE.

**Figure 5:**
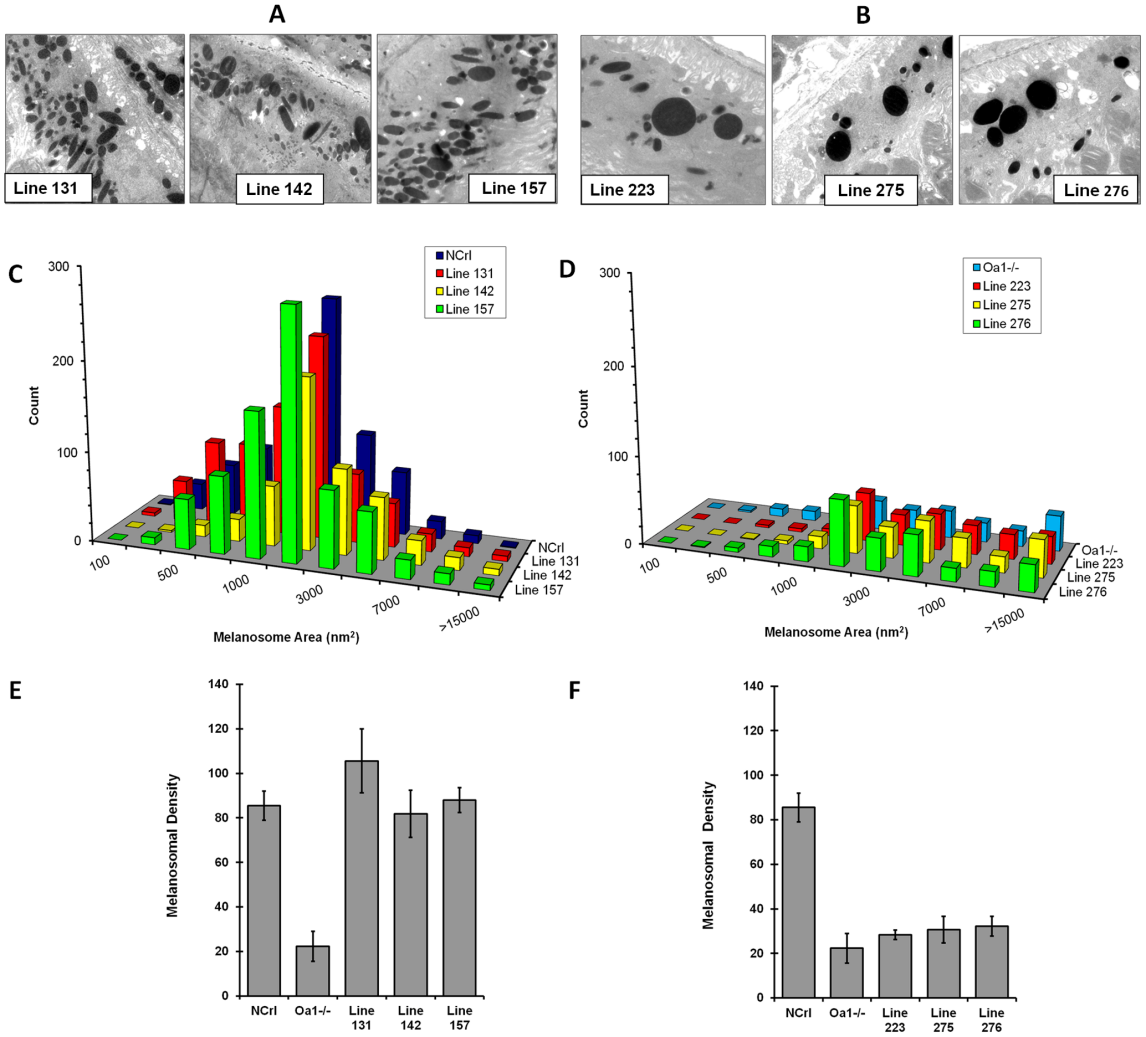
Electron microscopy and densitometry analysis of the progeny of transgenic founders, comparing the RPEs of strong positive transgenic Gαi3 (Q204L) animals and those of mice without integrated transgene. A. Electron micrographs of RPEs from positive-strong transgenic mice lines 131, 142, and 157. B. Electron micrographs of RPEs from mice without integrated transgene lines 223, 275, and 276. C and D. Histograms representing the percent of RPE melanosomal size distribution in transgenic mice and animals without integrated transgene, respectively. E and F. Melanosomal density analysis of transgenic and non-transgenic progeny, respectively.

Genotyping and protein expression studies of the progeny of founder transgenic mice showed that transgenic lines also could be divided into two expressions groups, high and low. Therefore, we analyzed the RPE morphology of the progeny with high transgene expression to determine whether the RPE melanosomal phenotype was restored similarly to that in founder line 16. Electron microscopy analysis showed, that the population and distribution of melanosomes in the high transgene expression group are completely different from their parental *Oa1−/−,* and rather similar to *Oa1+/+* NCrl control. On the other hand, as expected, the negative transgenic mice retained the *Oa1−/−* RPE phenotype, showing the presence of macromelanosomes (Figure 5 A and B).

Furthermore, morphometrical analysis of RPE melanosomes in these groups showed that presence of the transgene correlated with a high number of medium and small melanosomes. Mice identified as lines 131, 142, and 157, showed highest frequency of melanosomes 2,000 nm^2^ in size (29.1%, 37.3% and 35.3%, respectively) and had very low percentages of melanosomes larger than 15,000 nm^2^: 0.8%, 1.4% and 0.8%, respectively (Figure 5C). Conversely, the group without an integrated transgenene had the highest percentage of macromelanosomes and lowest number of small and medium melanosomes. For example, mouse lines 223, 275, and 276 had 13.0%, 16.9%, and 11.8% melanosomes larger than 15,000 nm^2^, respectively (Figure 5D and Table S1). The mean RPE melanosomal density in high expression lines 131, 142 and 157 was 105.6±14.3, 81.9±10.6 and 87.9±5.6, respectively (Figure 5E). On the contrary, negative transgenic lines 223, 275, and 276 had a reduced number of melanosomes per RPE area, 28.3±2.1, 30.6±6.0 and 32.2±4.4 respectively, which resembles the characteristic *Oa1−/−* RPE melanosomal phenotype (Figure 5F). Thus, our results demonstrated that transgenic animals with high transgene expression showed complete rescue of the melanosomal phenotype.

**Figure 6:**
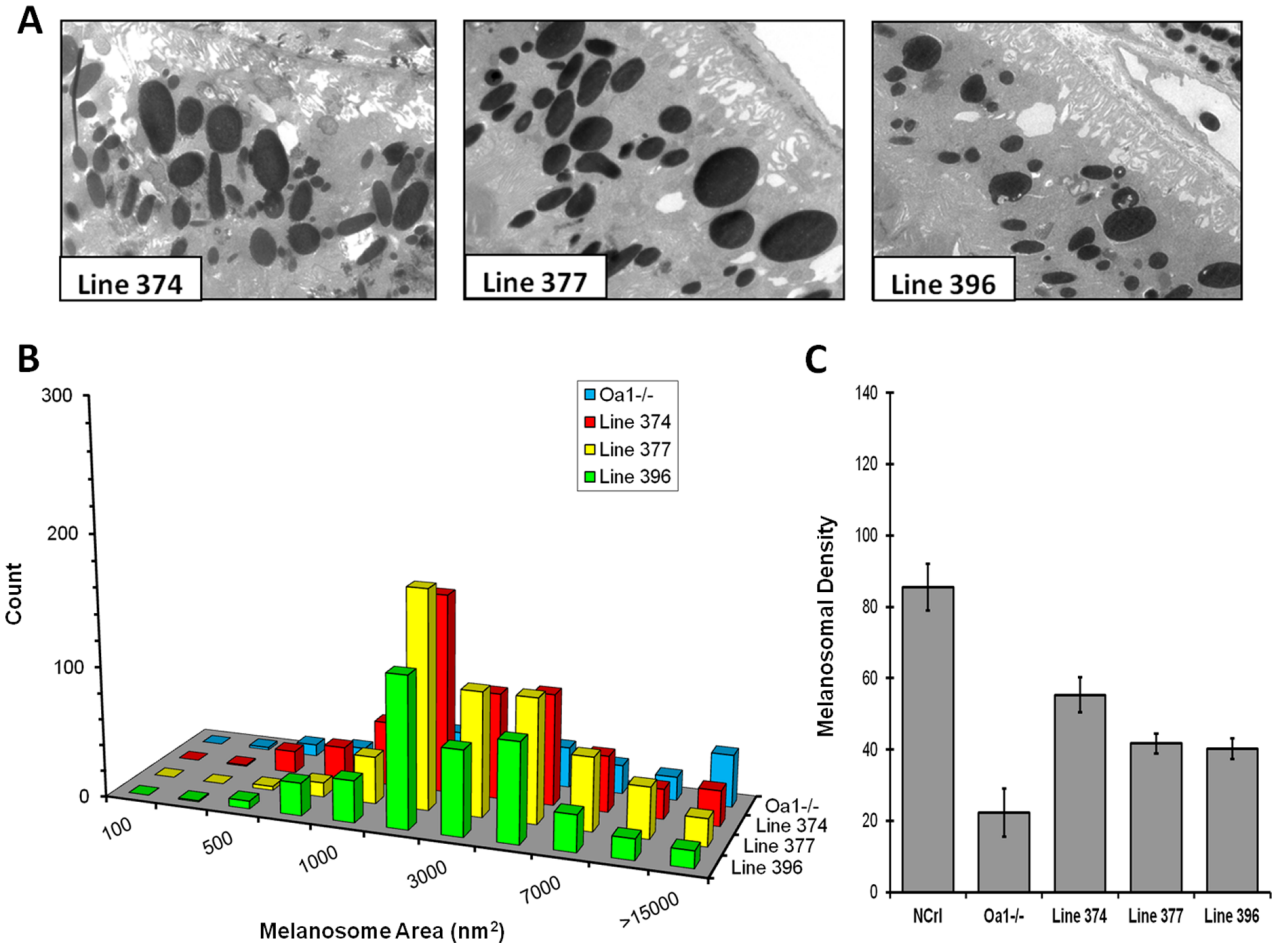
Electron microscopy of weak transgenic *Oa1−/−,* Gαi3 (Q204L) mouse RPEs and densitometry analysis of their melanosomes compared to those of wild-type NCrl and Oa1*−/−* mice. A. Electron micrograph of mouse RPEs from lines 374, 377, and 396. B. Histogram representing the percent distribution of melanosomal sizes. C. Melanosomal density analysis.

We also analyzed the transgenic lines with low transgene expression, which likely carry a single copy of the Gαi3 (Q204L) transgene. In these mice, a partial rescue of the RPE melanosomal phenotype was observed, with the presence of few macromelanosomes among many more small melanosomes (Figure 6A). Lines 374, 377, and 396 are examples of this type of mice that have 30.2%, 31.9%, and 30.5% melanosomes of 2,000 nm^2^ **,** respectively, and 5.4%, 4.0%, and 3.4% melanosomes of 15,000 nm^2^, respectively (Figure 6B). They also showed a partial rescue of the melanosomes per RPE area and presented a mean density of 55.3±4.9, 41.7±2.8, and 40.3±2.9, respectively (Figure 6C). This suggests there is a Gαi3 dose-dependent restoration for RPE melanogenesis.

In summary, our results demonstrate that the constitutively active Gαi3 protein indeed can rescue the abnormal RPE phenotype observed in *Oa1−/−* mice, by restoring the size and number of its melanosomes. This further corroborates the involvement of Gαi3 in RPE melanogenesis.

## Discussion

We have previously demonstrated by *in-vitro* and *in-vivo* studies on mice that Gαi3 physically interacts with the Oa1 protein in the RPE and that lack of Gαi3 from the mouse genome leads to the presence of RPE macromelanosomes and a reduced melanosomal density, suggesting that Gαi3 is the first downstream component in the Oa1 signaling pathway [10], [20]. This work was designed to test the hypothesis that Gαi3 is the major transducer for Oa1-mediated melanogenesis in RPE cells. We introduced a constitutively active Gαi3 protein in *Oa1−/−* RPE cells, to test i*n-vivo* if the activation of Gαi3 and its downstream signaling could bypass the lack of Oa1 protein and keep the cascade going, leading to normal pigmentation and melanosomal morphology of the RPE.

Transgenic mice were efficiently generated by infecting *Oa1−/−* fertilized eggs with a lentiviral vector carrying the constitutively active Gαi3 protein driven by the RPE-specific promoter of the *Oa1* gene. Substitution of a conserved glutamine for a leucine residue (Q204L) within the GTPase domain of Gαi3, results in a constitutively active form of this protein. A lentiviral vector was chosen to generate the transgenic animals, since it is able to transduce efficiently both dividing and non dividing cells [23]. Moreover, it has been shown that the transgenes delivered by lentiviral vectors are capable of escaping gene silencing and expressing stably *in-vivo* [24], [25]. The lentiviral construct contained not only the Gαi3 (Q204L) cDNA, but also the ires-GFP cDNA, which allowed us to determine the expression pattern and levels of the transgene.

After generating *Oa1−/−*, Gαi3 (Q204L) transgenic positive founder mice, we demonstrated the germline transmission of the transgene among their progeny. Our results showed that the presence of the constitutively active Gαi3 in the *Oa1−/−* RPE rescued the size distribution and density of its melanosomes. Most interestingly, the data showed that there is a strong correlation between the degree of rescue observed and the relative transgene expression level. Positive mice with a high expression level of the transgene presented an RPE with an average number of melanosomes (727±8.1) similar to that of the wild-type NCrl control mice (743±2.6), indicating that a high level of expression of the constitutively active Gαi3 (Q204L) is most likely sufficient to activate a normal melanogenesis signaling pathway. In contrast, transgenic mice with weak Gαi3 (Q204L) expression showed an increased average number of melanosomes (506±4.1) from that of Oa1*−/−* RPE (209±1.8) but still presented some macromelanosomes. This corroborated our hypothesis that there might be a necessary threshold of activated Gαi3 protein needed for proper melanosome biogenesis. The precise mechanism for regulation of the number of melanosomes in the RPE remains to be determined. It is noteworthy that transgenic founder mice and their progeny from interbreeding that have multiple copies of the transgene exhibit full restoration of the RPE phenotype while those transgenic founders or their offspring with a single copy showed partial restoration. This suggests that the melanosomal rescue observed in individual transgenic lines correlates simply to copy number rather than to the integration site in the genome of the transgenic founders.

Our data once again confirmed that Gαi3 is indeed the major transducer of Oa1 in this G-protein coupled receptor signaling cascade as well as its importance in controlling the regulation of RPE pigmentation. Furthermore, it depicted Gαi3 as the lead factor in melanogenesis, since its constitutive activation when the Oa1 receptor is not functional results in a normal signaling pathway. Thus, Gαi3 (Q204L) ensures the proper recruitment of melanosome biogenesis factors needed for the normal regulation of the size and number of RPE melanosomes. We previously proposed a model suggesting that Gαi3 controls the size of melanosomes in the RPE through the inhibition of vesicle trafficking from the trans-Golgi network (TGN) to the melanosomes [20]. This could explain the changes in RPE phenotype observed in ocular albinism when a non-functional OA1 protein (that cannot activate Gαi3) leads to a continuous vesicular traffic of membrane proteins to melanosomes resulting in the formation of macromelanosomes. This further supports the notion that giant melanosomes are formed by overgrowth of single melanosomes rather than by fusion of several small melanosomes. None of the electronmicrographs that we analyzed had melanosomes in the process of merging or dividing. Therefore, we conclude that there is no fusion or fission of these organelles during melanogenesis.

It has been shown that both α and βγ subunits of heterotrimeric G proteins are involved in the receptor signaling regulating many biological processes [26]. Interestingly, our data suggest that melanogenesis signaling is mediated mainly by Gαi3 alone, and that the Gi βγ moiety plays little or no role at all.

RPE abnormal pigmentation has been shown to be closely related to the abnormal routing of the optic axons [27], [28], [29]. An important question that remains to be address is whether the introduction of the constitutively active Gαi3 protein into the RPE rescues the misrouting of RGC axons at the *Oa1−/−* transgenic mice optic chiasm. If that were the case, we would be able to conclude that Gαi3, helping to maintain a healthy melanized RPE, is needed for proper development of the optic axon projections.

In summary, we have proved that a constitutively active Gαi3 protein circumvents the need for a functional Oa1 receptor to regulate RPE melanogenesis, and have confirmed that Gαi3 is essential in the Oa1 signaling cascade. The discovery of other members of this pathway will increase our understanding of ocular albinism and might open possibilities to develop therapeutic strategies for this disease.

## Supporting Information

Table S1 **Percentage of melanosomes by size distribution in transgenic lines, control wild-type, and** ***Oa1−/−*** **RPEs.** Each percentage was calculated from the total number of melanosomes in 12 electronmicrographs analyzed/sample, as described in Materials and Methods.
(DOC)
