# Supplementary material for: A Constitutively Active Gαi3 Protein Corrects the Abnormal Retinal Pigment Epithelium Phenotype of Oa1−/− mice
Source: PLoS One. 2013 Sep 30;8(9):e76240. doi: 10.1371/journal.pone.0076240 (PMC3787026; doi:10.1371/journal.pone.0076240)
Supplement: Table S1 — Percentage of melanosomes by size distribution in transgenic lines, control wild-type, and Oa1−/− RPEs. Each percentage was calculated from the total number of melanosomes in 12 electronmicrographs analyzed/sample, as described in Materials and Methods. (DOC) [file pone.0076240.s001.doc]

**Table S1:** Percentage of melanosomes by size distribution in transgenic lines, control wild-type, and *Oa1-/-* RPEs.

| **size nm^2^** | **NCrl** | **Oa1-/-** | **Line 16** | **Line 13** | **Line 131** | **Line 142** | **Line 157** | **Line 223** | **Line 275** | **Line 276** | **Line 374** | **Line 377** | **Line 396** |
| --- | --- | --- | --- | --- | --- | --- | --- | --- | --- | --- | --- | --- | --- |
| **100** | 0.1 | 0.0 | 0.1 | 0.0 | 0.5 | 0.0 | 0.0 | 0.0 | 0.0 | 0.0 | 0.0 | 0.0 | 0.0 |
| **300** | 4.0 | 1.0 | 0.4 | 0.0 | 6.0 | 0.4 | 1.0 | 0.0 | 0.0 | 0.4 | 0.2 | 0.0 | 0.3 |
| **500** | 7.7 | 4.3 | 3.3 | 0.0 | 12.3 | 2.6 | 7.2 | 1.3 | 0.4 | 2.0 | 3.4 | 0.6 | 1.6 |
| **700** | 10.9 | 5.3 | 11.7 | 1.6 | 12.6 | 4.9 | 11.1 | 1.7 | 1.6 | 4.7 | 5.0 | 2.1 | 6.6 |
| **1000** | 14.4 | 7.7 | 20.9 | 4.2 | 18.5 | 13.2 | 20.7 | 3.4 | 5.6 | 6.7 | 9.7 | 6.9 | 8.5 |
| **2000** | 34.6 | 15.8 | 45.7 | 18.8 | 29.1 | 37.3 | 35.3 | 23.1 | 21.8 | 29.1 | 30.2 | 31.9 | 30.5 |
| **3000** | 14.7 | 12.9 | 10.9 | 15.2 | 10.0 | 18.7 | 11.0 | 14.7 | 14.1 | 14.6 | 16.1 | 18.2 | 17.2 |
| **5000** | 9.6 | 14.8 | 4.9 | 18.8 | 6.4 | 13.6 | 8.7 | 17.2 | 19.0 | 18.1 | 16.9 | 18.2 | 20.2 |
| **7000** | 2.7 | 10.5 | 0.7 | 7.9 | 2.6 | 5.3 | 2.7 | 13.9 | 13.3 | 5.9 | 8.5 | 10.7 | 7.4 |
| **10000** | 1.2 | 8.6 | 1.0 | 6.8 | 1.3 | 2.8 | 1.6 | 11.8 | 7.3 | 6.7 | 4.6 | 7.5 | 4.2 |
| **>15000** | 0.1 | 19.1 | 0.4 | 26.7 | 0.8 | 1.4 | 0.8 | 13.0 | 16.9 | 11.8 | 5.4 | 4.0 | 3.4 |

Each percentage was calculated from the total number of melanosomes in 12 electronmicrographs analyzed/sample, as described in Materials and Methods.
